# Congenital Agenesis of the C6 Pedicle Leading to Misdiagnosis of a Traumatically Jumped Facet: A Case Report

**DOI:** 10.7759/cureus.1619

**Published:** 2017-08-28

**Authors:** Clay Elswick, Blake Walker, Marc Moisi, Vicki Diaz, Jeni Page, Justin Hugelier, Christian Fisahn, R. Shane Tubbs, Sussan Salas

**Affiliations:** 1 Neurosurgery, Wayne State University School of Medicine; 2 Neurosurgery, Seattle Science Foundation; 3 Neurosurgery, Swedish Neuroscience Institute; 4 Orthopedic Surgery, Swedish Neuroscience Institute

**Keywords:** cervical spine trauma, agenesis of cervical pedicle, spine trauma, cervical spine injury, cervical spine

## Abstract

This case report discusses the rare issue of an atrophic cervical pedicle at the C6 level in a patient found unconscious with a jumped facet and an unknown mechanism of injury. A means to discern between traumatic jumped facets versus congenital anomalies is addressed, including missing pedicles, which is encountered at the C6 level in this case. A literature review revealed that the most common level where this occurs is at the C6 level. The structural anatomic pathologies and the variants relative to congenital facet atrophy are identified, including the location and the surrounding vasculature; more specifically, the vertebral arteries. This information is helpful to assist clinicians when discerning between a traumatic subluxation injury that requires instrumentation and reduction versus a congenital anomaly that can usually be managed conservatively.

## Introduction

The evaluation of the cervical spine is a critical aspect of every trauma survey. Appropriate radiological studies must be ordered to determine if an injury exists and if surgical intervention is necessary. The severity of the injury will dictate if a cervical X-ray and a computed tomography (CT) scan are sufficient or further imaging with magnetic resonance imaging (MRI) is necessary [1].

Herein, we present a trauma case that was initially thought to have an acute traumatically jumped facet. However, after further evaluation, it was found to be a congenital anomaly. This case and germane literature are reviewed.

## Case presentation

**Figure 1:**
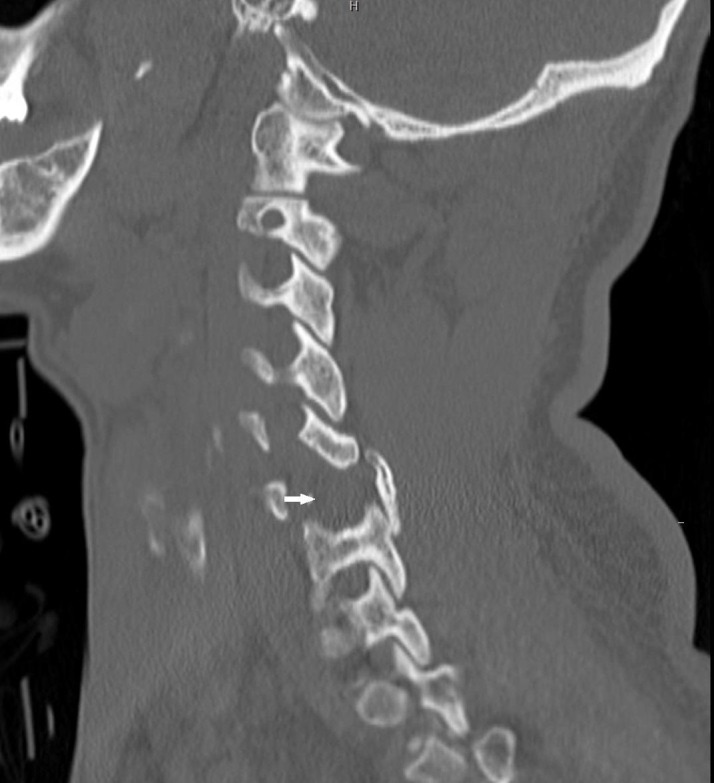
Computed Tomography Scan of the Cervical Spine Sagittal computed tomography scan of the cervical spine showing the missing C6 pedicle on the left side (white arrow)

**Figure 2:**
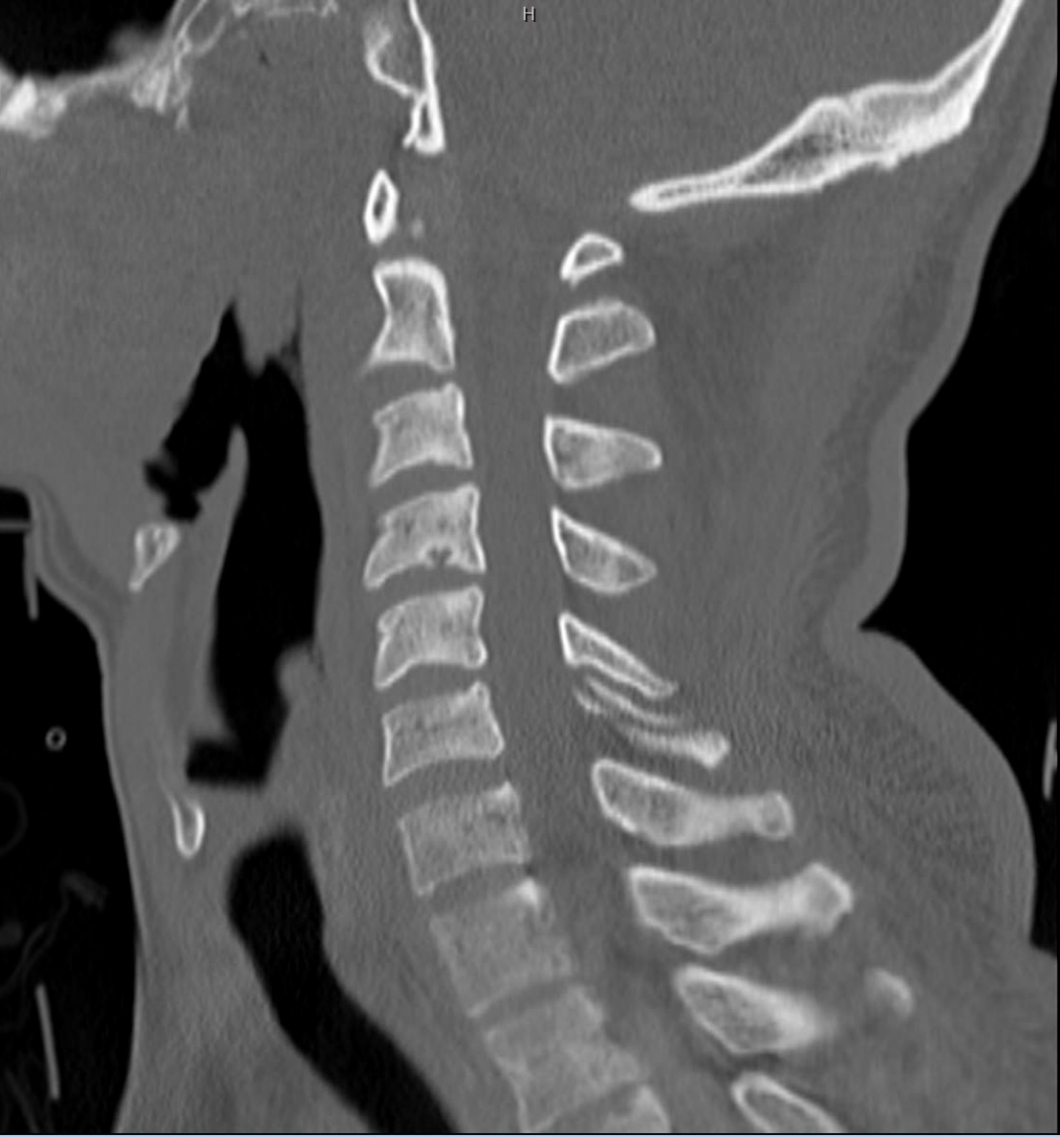
Computed Tomography Scan of the Cervical Spine Midline sagittal imaging that shows a lack of anterolisthesis

A 44-year-old man presented to the emergency department (ED) after being found unconscious in his bathroom. The sink was broken, with blood reported all over the bathroom, suggesting some form of traumatic fall in the bathroom. On arrival at the emergency department, he was found to have an altered mental status with several facial lacerations, and a head CT demonstrated a small amount of frontal subarachnoid hemorrhage and a focal right frontal intraparenchymal contusion. Furthermore, his blood glucose was nearly 700 mg/dl and he was in diabetic ketoacidosis (DKA). A neurosurgical evaluation of the patient revealed a Glasgow Coma Scale (GCS) score of 11 likely related to a combination of the DKA and the cerebral contusions. When he became cooperative, he was moving all extremities symmetrically. Given a suspicion for traumatic injury, a cervical collar was placed in the field and a CT C-spine was performed after arrival to the ED. The initial report of the CT described a right unilateral jumped cervical facet with a laminar fracture without subluxation shown in the sagittal in Figure 1. However, as demonstrated in Figure 2, the midline sagittal CT scan of the cervical spine did not show any anterolisthesis of C6. Therefore, a jumped facet is less likely.

**Figure 3:**
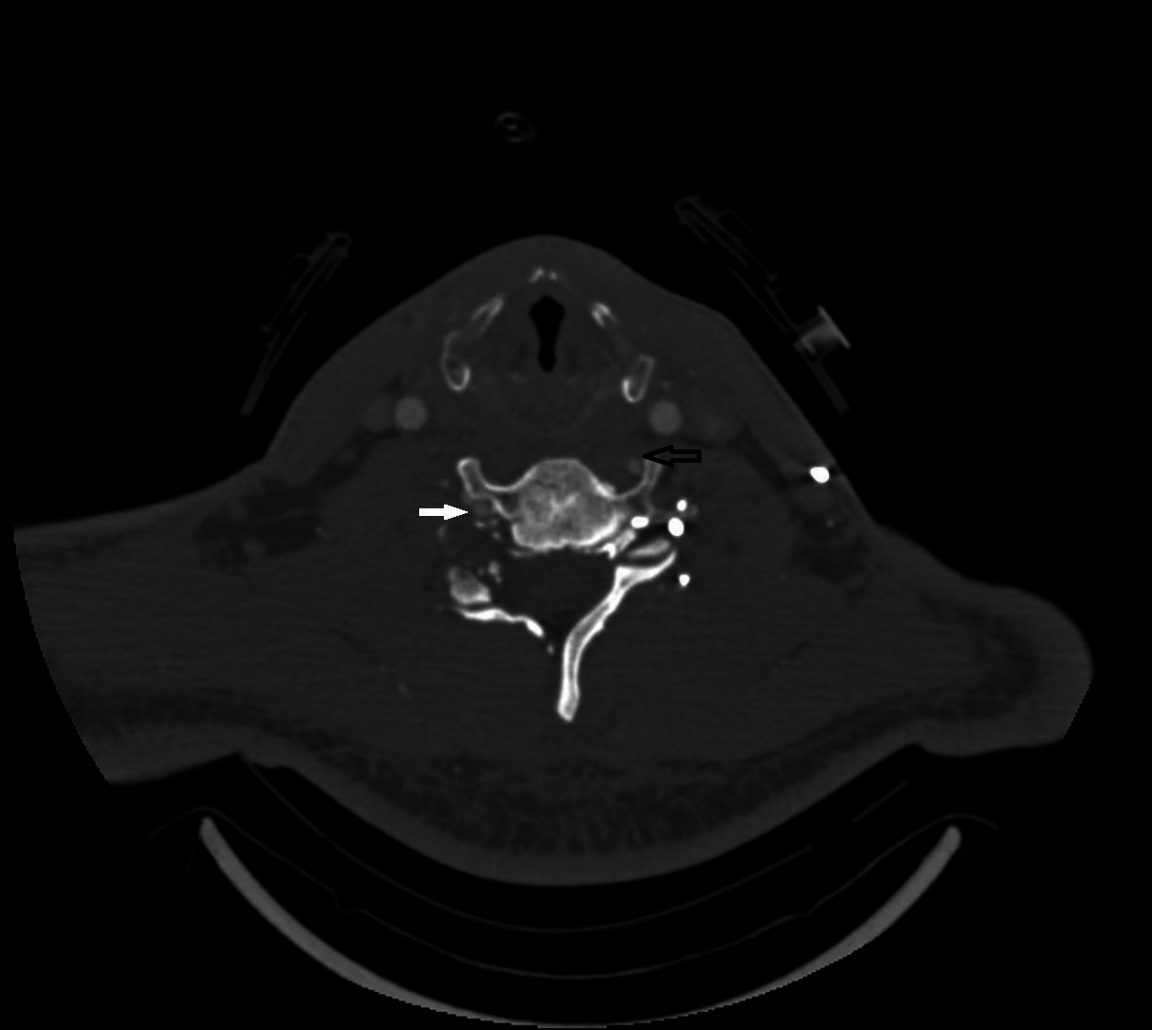
Computed Tomography Angiogram at C6 Computed tomography angiogram at the C6 level showing the vertebral artery in the foramen transversarium on the right (white arrow) and on the transverse process out of the foramen on the left (black arrow)

On closer inspection, however, it was evident that there was no pedicle on the right side at C6, and there was significant sclerosis around the edges of the lamina where the bone did not unite. A CT angiography (CTA) of the cervical spine demonstrated an entry point of the vertebral artery on the left side at the transverse foramen of C5. Figure 3 demonstrates the CTA at the level of C6 with the right vertebral artery already in the foramen transversarium while the left is still not in the foramen.

**Figure 4:**
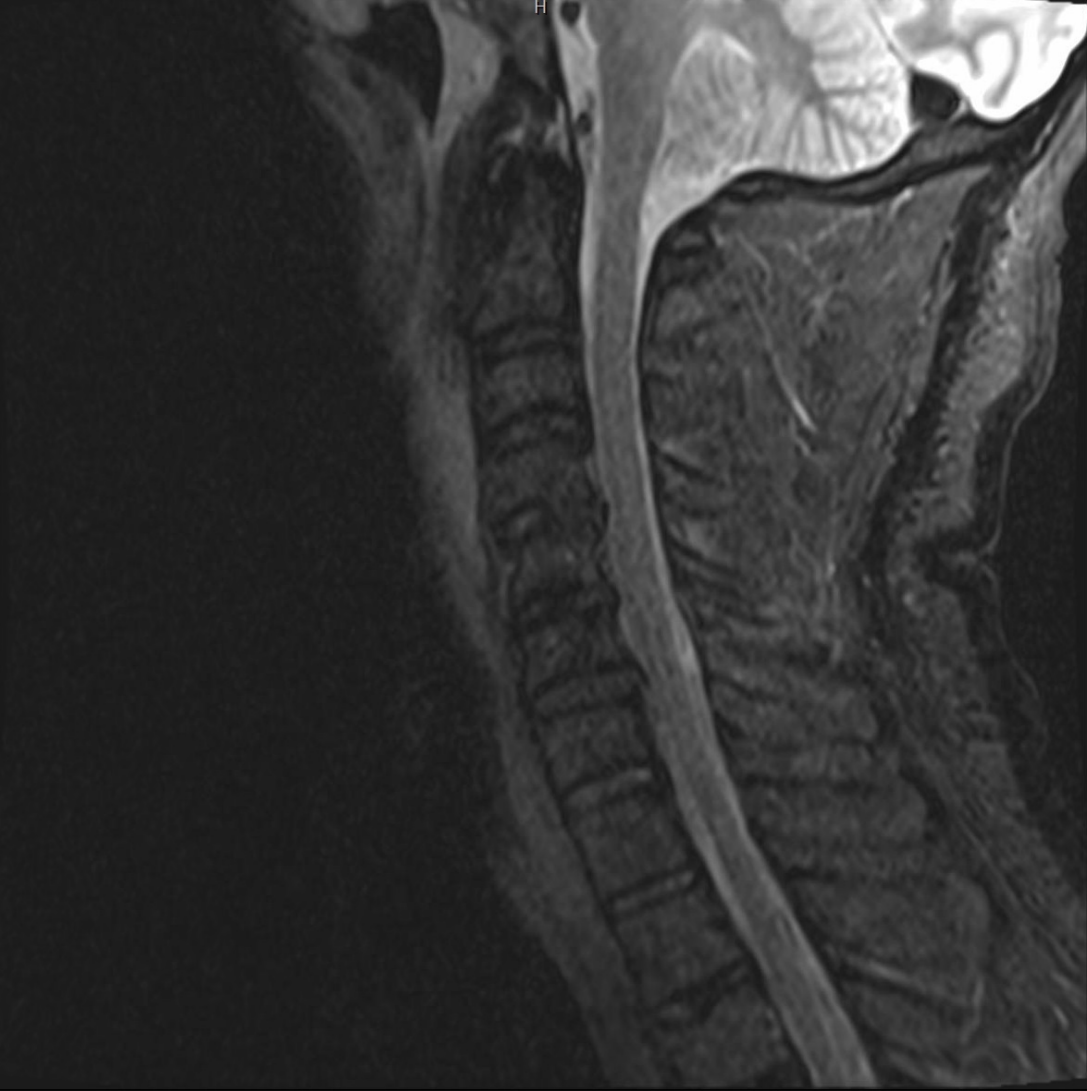
Magnetic Resonance Imaging of the Cervical Spine Sagittal cervical spine short tau inversion recovery (STIR) sequence

An MRI was also obtained, and there was no evidence of short tau inversion recovery (STIR) changes to suggest an acute injury, as shown in Figure 4.

## Discussion

A review of the literature identified reports of congenital spinal anomalies, including the absence of cervical pedicles. The earliest description in the medical literature of the congenital absence of a cervical pedicle was in 1946 [2]. There are just over 70 reports of this clinical entity in the medical literature since the original description [2]. In these cases, patients often present with some degree of neck pain following a traumatic event. Some patients also complain of paresthesias, but significant motor deficits are uncommon. Conservative treatment is usually recommended in the majority of the published cases and results in a resolution of the symptoms most of the time [3-6]. In these reports, the congenital anomalies are often misdiagnosed as traumatic subluxations. Radiographs suggest a traumatic subluxation with facet dislocation, and when this is seen, a CT scan with 3-D reconstructions should be obtained. There are multiple features that may help distinguish these congenital anomalies from traumatic fractures and include less soft tissue swelling and a lower degree of subluxation [3]. CT imaging with reconstructions demonstrates an apparently enlarged neural foramen on the side of the absent pedicle, a dorsally displaced articular pillar and lamina, and a dysplastic transverse process on the ipsilateral side [4]. The most common level where this occurs is C6, and the second-most common level is C5 [4]. Misdiagnosis may result in inappropriate treatment and an attempted reduction of the apparent subluxation. Moreover, when congenital anomalies, such as absent cervical pedicles, are diagnosed, one should also be aware that there might be variation in the origin, course, and entrance of the vertebral artery into the transverse foramen.

The course of the vertebral artery has been studied and described extensively in the medical literature. The vertebral artery is divided into four segments (V1-4). The V1 segment is from the origin to the foramen transversarium. The V2 segment is once it enters the foramen transversarium until it reaches the C2 foramen transversarium. V3 is from C2 to the dura and V4 is once the artery is intradural. The majority of the literature pertaining to spinal surgery focuses on the V3 segment of the vertebral artery and surgical pitfalls with respect to bony anatomy and aberrant arterial course at the C1-2 levels [5-6]. The level of the entrance of the vertebral artery into the transverse foramen is also a well-described topic in the medical literature. The clinical relevance to this relates to a safe dissection during an anterior cervical approach or undermining the longus colli muscle. It also relates to the placement of pedicle screws in the lower sub-axial spine and the safety of the placement of such instrumentation. In 2006, Bruneau et al. published the anatomical variations of the V2 segment of the vertebral artery based on 200 MRI studies and 50 CTAs (a total evaluation of 500 vertebral arteries). The vertebral artery entered the transverse foramen of C6 in 93% of all patients. An abnormal entrance was encountered seven percent of the time. The vertebral artery entered at C3 in 0.2% of cases, at C4 in 1%, at C5 in 5%, and at C7 in 0.8% [7].

## Conclusions

Congenital anomalies of the spine, though not common, may be misdiagnosed, especially in the setting of trauma. CT imaging in conjunction with an MRI scan with STIR imaging can reduce the probability of misdiagnosis. These anomalies might also be associated with an aberrant course of the vertebral artery, placing it at greater risk of injury during surgery. In this instance, a CTA of the neck was exceptionally useful to closely follow the course of the vertebral artery if surgical intervention was to be pursued.
